# Engineered Nanostructured Materials for Ofloxacin Delivery

**DOI:** 10.3389/fchem.2018.00554

**Published:** 2018-11-27

**Authors:** Silvia Nuti, Javier Fernández-Lodeiro, Benedetta Del Secco, Enrico Rampazzo, Benito Rodríguez-González, José L. Capelo, Vanessa Silva, Gilberto Igrejas, Patrícia Poeta, Cármen Torres, Nelsi Zaccheroni, Luca Prodi, Elisabete Oliveira, Carlos Lodeiro

**Affiliations:** ^1^BIOSCOPE Group, LAQV@REQUIMTE, Chemistry Department, Faculty of Science and Technology, NOVA University of Lisbon, Almada, Portugal; ^2^G. Ciamician Department of Chemistry, University of Bologna, Bologna, Italy; ^3^Proteomass Scientific Society, Costa de Caparica, Portugal; ^4^Scientific and Technological Research Assistance Centre (CACTI), University of Vigo, Vigo, Spain; ^5^Department of Genetics and Biotechnology, University of Trás-os-Montes and Alto Douro, Vila Real, Portugal; ^6^Functional Genomics and Proteomics Unit, University of Trás-os-Montes and Alto Douro, Vila Real, Portugal; ^7^Veterinary Science Department, University of Trás-os-Montes and Alto Douro, Vila Real, Portugal; ^8^LAQV-REQUIMTE, Faculty of Science and Technology, Nova University of Lisbon, Lisbon, Portugal; ^9^Área de Bioquímica y Biología Molecular, Universidad de La Rioja, Logroño, Spain

**Keywords:** mesoporous silica nanoparticles, silver nanoparticles, ofloxacin, bacteria, antibiotics

## Abstract

Antibiotic resistance is emerging as a growing worldwide problem and finding solutions to this issue is becoming a new challenge for scientists. As the development of new drugs slowed down, advances in nanotechnology offer great opportunities, with the possibility of designing new systems for carrying, delivery and administration of drugs already in use. Engineered combinations of the synthetic, broad-spectrum antibiotic ofloxacin, rarely studied in this field, with different types of silver, mesoporous silica-based and Pluronic/silica-based nanoparticles have been explored. The nanocarriers as silver core@silica mesoporous (AgMSNPs) and dye-doped silica nanoparticles functionalized with ofloxacin were synthesized and their antibacterial properties studied against *S. aureus* and *E. coli*. The best antibacterial results were obtained for the AgMSNPs nanosystem@ofloxacin for the strain *S. aureus* ATCC 25923, with MIC and MBC values of 5 and 25 μg/mL, proving the efficacy and synergetic effect of the antibiotic and the Ag core of the nanoparticles.

## Introduction

Antibiotics are one of the most important discoveries in modern medicine, because of their significant contribution in reducing the mortality and morbidity determined by infectious diseases. In the years ranging from 1945 to 1960 a lot of new antibiotics, still in use, were discovered and characterized (Wright, 2007).

Massive and often indiscriminate use of antibiotics has led to a dramatic increase in manifestations of antibiotic resistance (Wright, 2007). Antibiotic resistance is present for example in hospitals, where significant quantities of drugs are used, or in countries where antibiotics were available without a prescription or medical advice (Levy and Marshall, 2004; Andersson and Hughes, 2010). Moreover, the use of sub-lethal doses of an antibiotic can end up in manifestations of multidrug resistance (MDR) (Kohanski et al., 2010). The most common bacterial infections that require hospitalization are caused by *Staphylococcus aureus* (*S. aureus*), followed by *Escherichia coli* (*E. coli*) (Sen Karaman et al., 2016).

Bacteria can develop antibiotic resistance with a variety of mechanisms such as horizontal gene transfer mechanism, which enables bacteria to “share” resistant gene sequences (Davies, 1994).

Bactericidal antibiotics as quinolones are often used as last chance when no other therapy is effective, but they can induce bacteria to produce reactive oxygen species (ROS) (Dwyer et al., 2007), causing DNA damage and mutations accumulation (Kohanski et al., 2010).

Antibiotic resistance poses several problems regarding public health, and it lowers the possibility of treating the infection and increases the possibility of a deadly outcome.

Aware of this problem, there is an increased need for the development of new platforms for overcoming antibiotic resistance (Pelgrift and Friedman, 2013). Antibacterial nanomaterials have been proved to be very efficient against antimicrobial resistance, allowing effective delivery of the antibiotics (Pelgrift and Friedman, 2013). In this framework, many different nanomaterials have been investigated and reported as carrier for antibiotics, in particular, metal-based nanocarriers (silver, zinc, titanium and gold nanoparticles), polymeric and silica nanoparticles. However, the synthesis and preparation of some of these nanocarriers could represent a drawback being more cost-effective than that of antibiotics (Zhang et al., 2010; Li et al., 2011; Tang et al., 2012; Webster and Seil, 2012; Bagga et al., 2017).

In this paper, we present a study on the combination of the most promising nanocarriers (silver and silica-based ones), for ofloxacin loading and release against Gram-positive and Gram-negative bacteria.

Silver nanoparticles (AgNPs) are known for their broader spectrum of antibacterial activity, in comparison to other metal nanoparticles, but they can undergo degradation and slow release of silver ions. It is possible, however, to prevent this inconvenience by incorporating them within silica nanoparticles, leading to more effective nanocarriers with a wider range of antibacterial applications than bare AgNPs (Guzman et al., 2012; Le Ouay and Stellacci, 2015; Tang and Zheng, 2018).

We chose to use the antibiotic ofloxacin (9-fluoro-3-methyl- 10-(4-methyl-1 -piperazinyl)- 7-oxo-2, 3-dihydro-7H-pyrido-(1, 2, 3-de)l, 4-benzoxazine-6-carboxylic) in our studies since it is active against Gram-positive, Gram-negative, and anaerobes bacteria (Sato et al., 1982). The activity of ofloxacin against *Enterobacteriaceae, Pseudomonas aeruginosa, Haemophilus influenzae, Neisseria gonorrhoeae, and Clostridium perfringens* is comparable to that of norfloxacin and far exceeds that of pipemidic and nalidixic acids. Some Gram-positive bacteria belonging to *Staphylococcus* spp. and *Streptococcus* spp., which are resistant to nalidixic acid, are instead susceptible to norfloxacin and ofloxacin, which provide a better bactericidal activity (Sato et al., 1982).

Recently Ding et al. reported on the antibacterial activity against two strains of *Pseudomonas aeruginosa* (WT and ΔABM) of AgNPs with three different sizes and all covalently functionalized with ofloxacin (Ding et al., 2018). They found that the inhibitory effect of ofloxacin is highly dependent on the concentration and size of the nanocarrier. The lowest MIC (Minimum Inhibitory Concentration) values (0.11 ± 0.01 μM for WT and 0.010 ± 0.001 μM for ΔABM) were obtained for the largest nanocarrier conjugated with 6.5 × 10^5^ ofloxacin molecules/nanoparticle against the free ofloxacin [0.59 ± 0.16 μM for WT and 0.096 ± 0.096 μM for ΔABM; (Ding et al., 2018)].

Despite the tremendous antibacterial properties of AgNPs, silica nanoparticles (SNPs) are also very promising nanomaterials due to their versatility, chemical and thermal stability (He and Shi, 2011; Tang et al., 2012). Silica nanoparticles and in particular mesoporous silica nanoparticles (MSNPs), in fact, are very often applied in the biomedical field, both in diagnosis and therapeutics, while examples of their use as antibacterial agents are much more seldom (Tang et al., 2012). High surface and pore volume of MSNPs allow the loading of several antibiotics, leaving their surface free and adaptable for a better cell internalization, leading to the creation of a new generation of antibacterial agents with improved synergistic effects (Tang et al., 2012). Moreover, surface functionalization allows better control of antibiotic release (Bhattacharyya et al., 2012). Recently, an antibacterial study of mesoporous silica nanoparticles with silver ion doping and chitosan surface coating was carried out against *E. coli*, and *V. cholera* and an efficacy improvement were achieved by the synergistic antibacterial effect of MSNPs combined with kanamycin (Sen Karaman et al., 2016). To the best of our knowledge, we present here for the first-time silver core @ silica mesoporous and dye-doped silica nanocarriers functionalized with ofloxacin, as well as, the study of their antibacterial properties against *S. aureus* and *E. coli*.

## Materials and methods

### Chemicals

1,6-diaminohexane (98%), Acetic Acid (HOAc ≥ 99.7%), Acetone, Borate Buffered Saline (tablets), Dichloromethane (DCM, reagent grade), Diethyl Ether, Dimethylformamide (DMF, reagent grade), Ethyl Ether (reagent grade), Hydrochloric Acid (≥37%), Hydrogen Peroxide (30%), N-Hydroxysulfosuccinimide sodium salt (NHS-Sulfo), N-N'- disuccinimidyl carbonate (DSC, ≥98%), Ofloxacin, Phosphate Buffered Saline (tablets), Pluronic F-127, Rhodamine B base (≥98%), Silver Nitrate (99%), Sodium Borohydride (99%), Sodium Citrate tribasic dehydrate (99%), Tetraethyl Orthosilicate (TEOS, 99.99%), Tetrahydrofuran (THF), Trimethylchlorosilane (TMSCl, ≥98%), Toluene were purchased from Sigma Aldrich. 3-(triethoxysilyl)propyl isocyanate (≥95%), Sodium Chloride, Piperazine (≥98%), Triethylamine (≥99.5 %) were purchased from Fluka. Absolute Ethanol, Dimethyl Sulfoxide (DMSO), Ethylene Glycol were purchased from Carlo Erba Reagents. Ammonium Nitrate, Hexadecyltrimethylammonium bromide (CTAB, 98%) were purchased from Alfa Aesar. Potassium Bromide and Sodium Hydroxide were purchased from Panreac. All reagents were used without further purification.

### Instrumentation and characterization techniques

Absorption spectra were acquired using the spectrophotometers JASCO V-630, JASCO V-650, and emission and excitation spectra were acquired using a spectrofluorometer Horiba-Jobin-Yvon Flouromax-4, from the PROTEOMASS-BIOSCOPE Facility at the Chemistry Department of the FCT-UNL, Portugal, and a spectrofluorometer Perkin Elmer LS55 (University of Bologna—Italy). The size, size distribution and the zeta potential of the nanoparticles were evaluated via Dynamic Light Scattering (DLS) using a Malvern Zetasizer Nano ZS equipped with a He-Ne laser source (633 nm, 5 mW), from the PROTEOMASS-BIOSCOPE Facility at the Chemistry Department of the FCT-UNL, Portugal.

Freeze drying of AgMSNPs with ofloxacin was achieved using a Christ Alpha 1-2 LD Plus, from the PROTEOMASS-BIOSCOPE Facility at the Chemistry Department of the FCT-UNL, Portugal. NPs were dried over 24 h.

TEM images were acquired using a JEOL JEM 1010 working at 100 kV at the Centro de Apoyo Científico y Tecnológico a la Investigación (CACTI), University of Vigo, Spain.

Scanning electron micrographs (SEM) were obtained with a high-resolution SEM-FIB microscope Zeiss Auriga CrossBeam System, CENIMAT—FCT-UNL.

The lifetime measurements were performed in a Horiba Jobin-Yvon Tempro, from the PROTEOMASS-BIOSCOPE Facility at the Chemistry Department of the FCT-UNL, Portugal.

Pore size distribution and surface area determination result from the measurement of N_2_ adsorption/desorption, at 77 K, in a Micromeritics ASAP 2010 (Accelerated Surface Area and Porosimetry), at the Laboratory of Analysis from FCT - UNL. The specific surface areas (S_BET_) were calculated from the adsorption data in the low pressures range using the BET model. Pore size was determined following the BJH (Barrett-Joyner-Halenda analysis) method.

The fluorescence quantum yield (Montalti et al., 2006) of dye-doped silica nanoparticles was measured using as standard rhodamine B (φ = 0.70) (Arbeloa et al., 1989) in ethanol and calculated using equation (1).

(1) $$\begin{array}{l} \Phi_{\text{S}}=\Phi_{\text{std}}\left(I_{\text{s}}/I_{\text{std}}\right){\left(n_{s}/n_{std}\right)}^{2} \end{array}$$

Where Φ_S_ and Φ_std_ are the radiative quantum yields of the sample and standard respectively, *n*_*s*_ and *n*_*std*_ are the refractive indexes (respectively, pure solvents were assumed), I_s_ and I_std_ the emission areas. The sample and the standard were excited at the same wavelength in an isosbestic point.

### Synthesis of nanoparticles

#### Synthesis of silver nanoparticles (AgNPs)

The AgNPs with spherical shape were synthesized using the method proposed by Frank et al. (2010). In a round bottom flask were added, 2.0 mL of a solution 1.25 × 10^−2^ M of sodium citrate, 5.0 mL of a solution 3.75 × 10^−4^ M of silver nitrate, 5.0 mL of a solution 5.0 × 10^−2^ M of hydrogen peroxide, 40 μL of a solution 1.0 × 10^−3^ M of potassium bromide in MilliQ water. The mixture was gently stirred for approximately 3 min, until a yellow color was observed. The solution of AgNPs was finally stored at 4°C.

#### Synthesis of mesoporous silica nanoparticles (MSNPs)

MSNPs were synthesized in aqueous media, using TEOS as silica precursor, according to the following protocol published by E. Oliveira et al. (2018). Briefly, 100 mg of CTAB (CH_3_(CH_2_)_15_N(Br)(CH_3_)_3_ were dissolved in 10 mL of H_2_O MilliQ, stirred and heated to about 50°C. To this solution were added 30 mL of H_2_O MilliQ, 10 mL of ethylene glycol and 157 μL of a 0.95 M aqueous solution of NaOH. This mixture was stirred at 70°C for 30 min, then 750 μL of TEOS were added drop wise and left stirring at 70°C for 3 more hours.

The template was removed by the addition of ammonium nitrate in ethanol at 60°C (Zhang et al., 2011). The final mesoporous nanoparticles were washed several times with water and ethanol and left to dry at room temperature (r.t.).

#### Synthesis of silver core- mesoporous silica shell nanoparticles (AgMSNPs)

For the synthesis of silver core-mesoporous silica shell nanoparticles, approximately 10 mL of AgNPs solution, obtained with the Frank method (Frank et al., 2010), were added in the first phase of the synthesis of mesoporous silica nanoparticles.

The template removal and the final washing were performed with a solution of NH_4_NO_3_ in EtOH.

##### PDMAC (poly(diallyldimethylammonium chloride) covering

The covering was applied on 10 mg of NPs using 20 μL of a PDMAC solution 10 mg/mL in a total volume of 2 mL of MilliQ water. After 2 h stirring at r.t. the solution was centrifuged and washed 7 times with 2 mL of MilliQ water (10,000 rpm, 15 minutes), then finally resuspended in 2 mL of MilliQ water. The size and zeta potential measurements were conducted on 0.5 mL of the previous solution diluted in 1.5 mL of MilliQ water.

The final mesoporous nanoparticles were obtained as a powder, and characterized by TEM, SEM, DLS, ICP and N_2_ isotherms.

#### Synthesis of dye-doped silica nanoparticles (SNPS)

Pluronic F127 derivatives, the triethoxysilane derivative of Rhodamine B and dye-doped silica NPs, presenting –NH_2_ functional groups (NPs-NH_2_**)**, were synthesized following procedures previously reported (Rampazzo et al., 2010, 2013). The final nanostructure was then conjugated to ofloxacin as described hereafter.

##### Synthesis of Pluronic F-127-amino

**Scheme 1:**
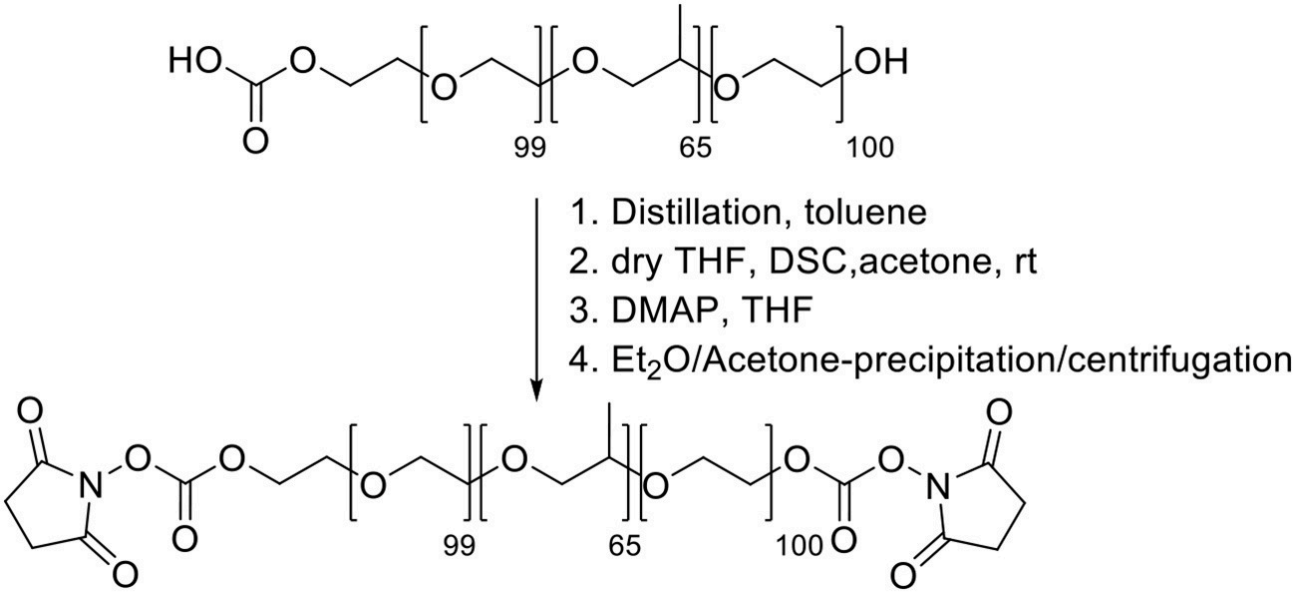
Synthesis of Pluronic F127-carbonate.

*Synthesis of Pluronic F-127-carbonate* Pluronic F127-carbonate was synthesized (Scheme 1) adapting reported procedures (Rampazzo et al., 2013). A toluene solution of Pluronic F127 (4.01 g, 100 mL) was distilled under reduced pressure at 50–60°C in a 250 mL round bottom flask. The residue was dried under vacuum and solubilized in 15 mL of dry THF. To this solution first 0.80 g of N,N'- disuccinimidyl carbonate was added in 7.0 mL of acetone and then 0.41 g of 4-(Dimethylamino)pyridine (DMAP, 99%) were slowly added by a dropping funnel at room temperature and under stirring. After 12 h, Pluronic F127-carbonate was precipitated with ethyl ether and recovered after circa 20 h of dialysis vs. MilliQ water, in regenerated cellulose dialysis tubing (Sigma, 12 kDa cut-off) under gentle stirring. The dialyzed solution was centrifugated at 7,000 rpm using 50 mL plastic falcon vials with acetone and finally dried under vacuum and directly used for amine functionalization.

**Scheme 2:**
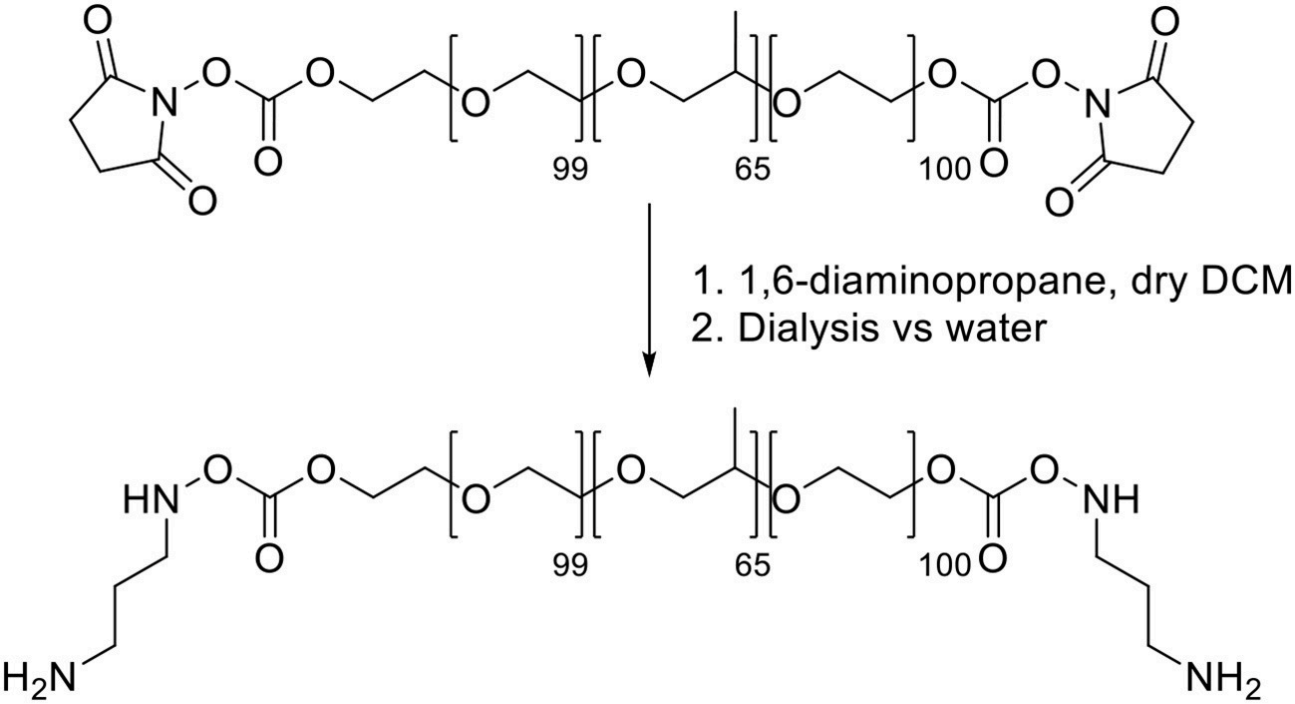
Synthesis of Pluronic F127-amino.

*Synthesis of Pluronic F-127-amino* Pluronic F127-amino was synthesized (Scheme 2) adapting reported procedures (Rampazzo et al., 2013). In a flamed 100 mL round bottom flask dried under vacuum, 1.9 g of 1,6-diaminopropane were solubilized with 10 mL of dry dichloromethane. 10 mL of a dichloromethane solution containing 2.0 g of F127-carbonate was then slowly added under stirring at room temperature. After 3 h the reaction mixture was distilled under reduced pressure and the residue was solubilized with 20 mL of water. The resulting solution was dialyzed vs. MilliQ water, for about 20 h, in regenerated cellulose dialysis tubing (Sigma, 12 kDa cut-off) under gentle stirring. The dialyzed solution was evaporated under reduced pressure and finally dried under vacuum.

##### Synthesis of the triethoxysilane derivative of rhodamine B (R)

**Scheme 3:**
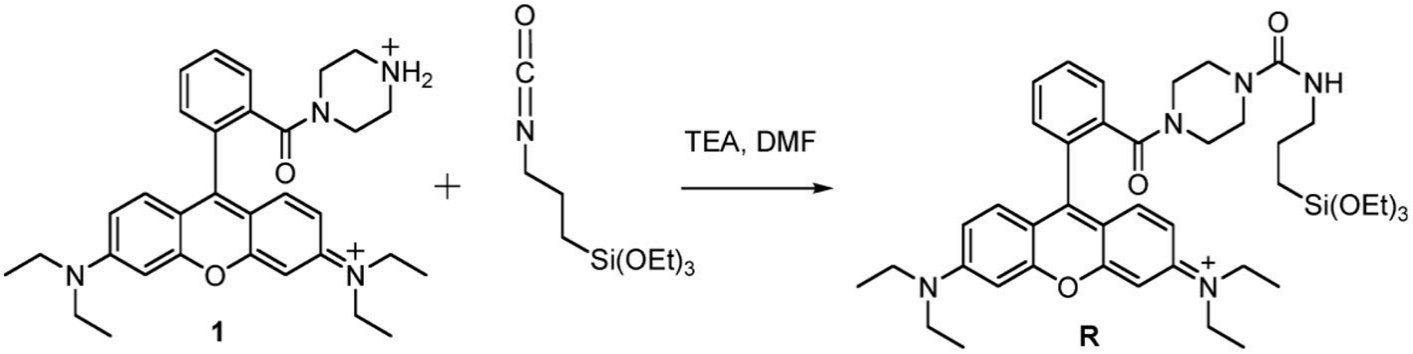
Synthesis of Silanized Rhodamine B.

*Synthesis of the tertiary amide from rhodamine B base* The tertiary amide **1** (Scheme 3) was obtained from Rhodamine B base following a previously reported procedure by Nguyen and Francis (Nguyen and Francis, 2003).

*Synthesis of the triethoxysilane derivative of rhodamine B (R)*. In a dried flask under N_2_ atmosphere, 500 mg of the tertiary amide **1** and 2.02 mL of triethylamine were dissolved in 7.5 mL of anhydrous DMF. 6.77 mL of 3-(triethoxysilyl)propyl isocyanatin in 10 mL of CH_2_Cl_2_ were added dropwise to this solution, at room temperature under stirring. After 12 h, the reaction mixture was dried under reduced pressure. We solubilized the obtained solid adding the minimal amount of EtOH and then we precipitated it by dropwise addition of a large volume (~300 mL) of Et_2_O. The resulting heterogeneous mixture was cooled under gentle stirring and the product was obtained by filtration under reduced pressure. The retained dark red-purple solid was rinsed with Et_2_O and recrystallized with EtOH/Et_2_O. The product **R** was obtained by filtration as a dark red-purple solid (Scheme 3).

##### Synthesis of dye-doped silica nanoparticles

**Scheme 4:**
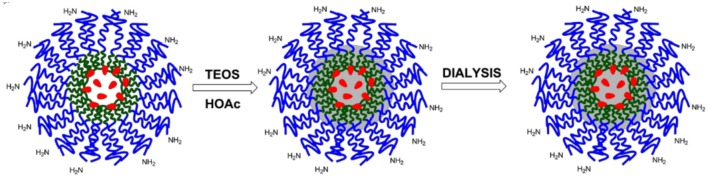
Schematization of the synthesis of SNPs-NH_2_.

Dye-doped silica NPs, presenting –NH_2_ functional groups at the surface were prepared following the synthesis sketched in Scheme 4. 140 mg of Pluronic F127, 60 mg of Pluronic F127-amino and 3.60 mg of the triethoxysilane derivative of Rhodamine B were carefully solubilized with 1 mL of dichloromethane in a 20 mL glass scintillation vial.

The solvent was evaporated from the homogeneous solution by means of a gentle nitrogen flow and subsequently under vacuum at room temperature. One hundred and thirty seven milligram of NaCl were subsequently added, and the mixture was solubilized at 30°C under magnetic stirring with 3.1 mL of CH_3_COOH 1M. 360 μL of TEOS were added to the solution and reacted for 3 h then 20 μL of TMSCl were added. The mixture was kept under stirring for 20 h at 30°C. The purification was carried out by dialysis vs. MilliQ water, during approximately 48 h, in regenerated cellulose dialysis tubing (Sigma, 12 kDa cut-off) at RT under gentle stirring (Montalti et al., 2014).

### Encapsulation and functionalization of ofloxacin into nanoparticles

The interaction of AgNPs with ofloxacin was studied by mean of spectrophotometric titrations and in batch mode for 24 h incubations.

Ofloxacin was encapsulated in MSNPs and AgMSNPs stirring for 24 h at room temperature and dark conditions. The encapsulation was conducted in DMSO for MSNPs and both in DMSO and PBS pH 7.4 for AgMSNPs.

The encapsulation percentage (%E) was evaluated after centrifuging NPs (MSNPs and AgMSNPs; 5 min, 10,000 rpm) and acquiring the absorption spectra of the supernatant. Encapsulation percentage (% E) final values were calculated through the following equation (2).

(2) $$\begin{array}{l} E\left(\%\right)=\frac{C_{i}-C_{e}}{C_{i}}\;\times \;100\% \end{array}$$

Where, C_i_ (M) is the initial concentration of ofloxacin, C_e_ (M) is the concentration of the ofloxacin in the supernatant.

**Scheme 5:**
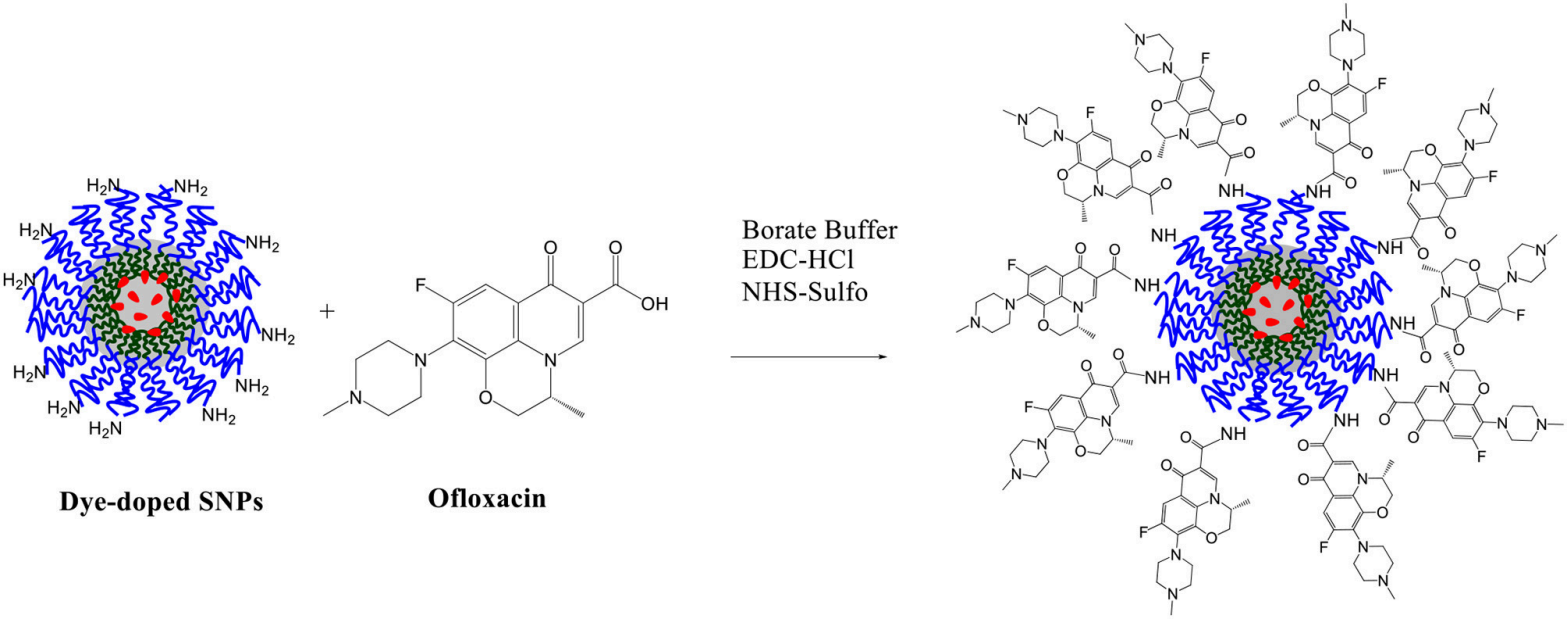
Functionalization of dye-doped SNPs with ofloxacin.

Ofloxacin was covalently linked to the dye-doped SNPs terminal amine groups according to the following protocol: a 0.03 M aqueous solution of ofloxacin was mixed with 304 μL of a 0.2 M aqueous solution of Sulfo-NHS and with 152 μL of a 0.2 M aqueous solution of EDC-HCl, this mixture was stirred for 30 min a room temperature with the aid of a vortex. To this mixture, we added 3.36 mL of a solution of NPs and the solution was stirred for 12 h at room temperature. The coupling procedure was carried out in 10.0 mL of borate buffer (0.05 M, pH 9.0; Scheme 5). The resulting solution was dialyzed vs. MilliQ water, for about 20 h (regenerated cellulose dialysis tubing Sigma, 10 kDa cut-off).

### Bactericidal activity test

#### Bacterial strains, culture media, and growth conditions

Bacterial strains considered in this study were *E. coli* C999 and *S. aureus* C5932 both resistant to ofloxacin, and the control strains *E. coli* ATCC 29425 and *S. aureus* ATCC 25923, both sensitive to ofloxacin (Table 1). The strains were supplied by the University of La Rioja and University of Trás-os-Montes and Alto Douro collection. All bacterial strains were grown in BHI agar (Oxoid, UK) for 24 h at 37°C.

**Table 1:** Bacterial strains used in this study.

**Strain**	**Relevant phenotype**	**Reference**
*E. coli* K12 ATCC 29425	Non-pathogenic, Gram-negative; sensitive to ofloxacin	ATCC
*E. coli* C999 (CTX-M-15)	Pathogenic, Gram-negative; resistant to ofloxacin	Ruiz et al., 2012
*S. aureus* ATCC 25923	Gram-positive, sensitive to ofloxacin	ATCC
*S. aureus* C5932 (MRSA CC398)	Non-pathogenic, Gram-positive, resistant to ofloxacin	Benito et al., 2014

#### Preparation of stock solutions

Each solution containing NPs (MSNPs, AgMSNPs, and Dye-doped SNPs) was diluted to final concentrations of 1, 10, 25, 50, 75, 100, 150, 200, 300, and 500 μg/mL in DMSO, and tested on all bacteria. Ofloxacin was tested in the range 1–500 μg/mL. As a control sample, the DMSO was also tested.

#### Antibacterial susceptibility test

The minimum inhibitory concentration (MIC), described as the lowest concentration of nanoparticles that inhibits the bacterial growth, was determined by broth-dilution method using a 96-well polystyrene microtiter plate. Luria-Bertani (LB) (Sigma–Adrich) broth was prepared and 135 μL were added to each well. Ten microliters of each solution containing different NPs with final concentrations ranging from 1 to 500 μg/mL were added to each well and 10 μL of overnight cultures of the selected bacteria were inoculated into the wells and incubated at 37°C. After 24 h, we measured the absorbance with a microplate spectrophotometer. The test was performed in triplicate. Positive and negative controls were performed using ofloxacin, Ag@MSNPs, MSNPs and DMSO.

To determine the minimum bactericidal concentration (MBC), which is characterized by no bacterial growth, 100 μL of the cultures resulting from MIC testing were inoculated onto LB medium plates and incubated at 37°C for 20 h. Control cultures without NPs were included in all experiments. The number of colony-forming units (CFUs) was determined by plate counting for each concentration and each sample.

## Results and discussion

### Synthesis of silver nanoparticles and interaction with ofloxacin

**Figure 1:**
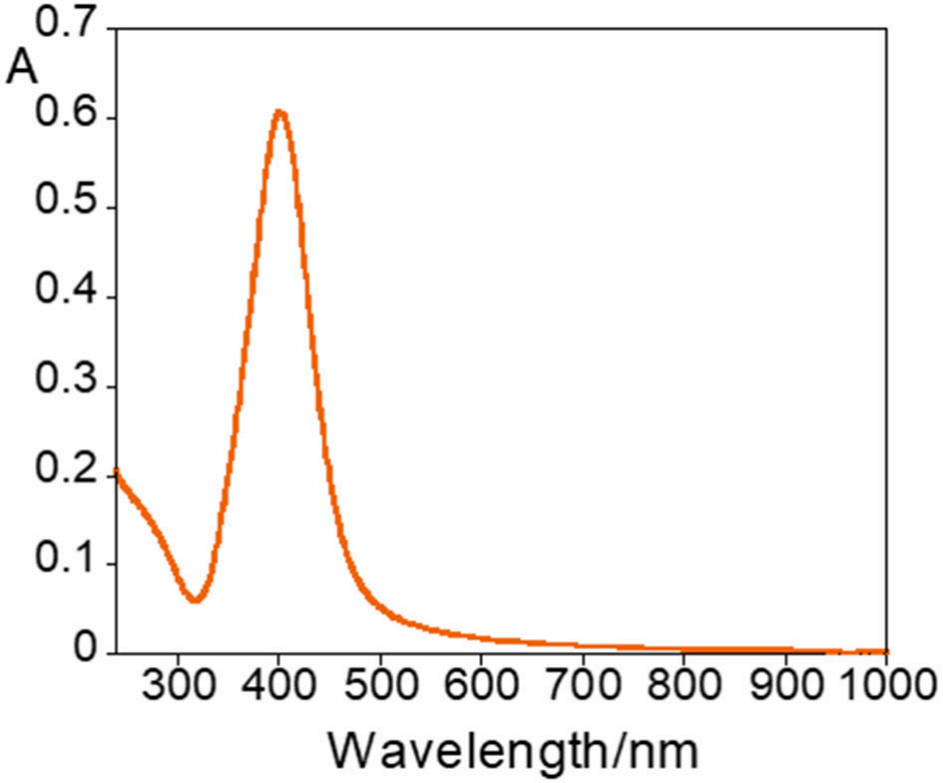
Absorption spectra of AgNPs.

Silver nanoparticles (AgNPs) were synthesized following the Frank method (Frank et al., 2010), and characterized by UV-Vis absorption measurements and DLS to determine their size and zeta potential. Diluting 1 mL of AgNPs obtained from the synthesis with 1.5 mL of MilliQ water a yellow solution was obtained with an absorption spectrum showing a plasmonic band at ca. 400 nm (Figure 1).

The average values of the hydrodynamic diameter, PDI and zeta potential were of 57 ± 30 nm, 0.3 ± 0.1 and −40 ± 4 mV, respectively, for *n* = 13 (*n* = number of replicates). In general, the AgNPs are very stable due to their high negative zeta potential.

The interaction with ofloxacin was initially studied by means of spectrophotometric titration. To 2.5 mL of aqueous AgNPs solution, obtained from the Frank method synthesis, a solution of 1 mg/mL of ofloxacin was added in 2 μL aliquots until a total of 78 μL was added, corresponding to a final concentration of 0.03 mg/mL

**Figure 2:**
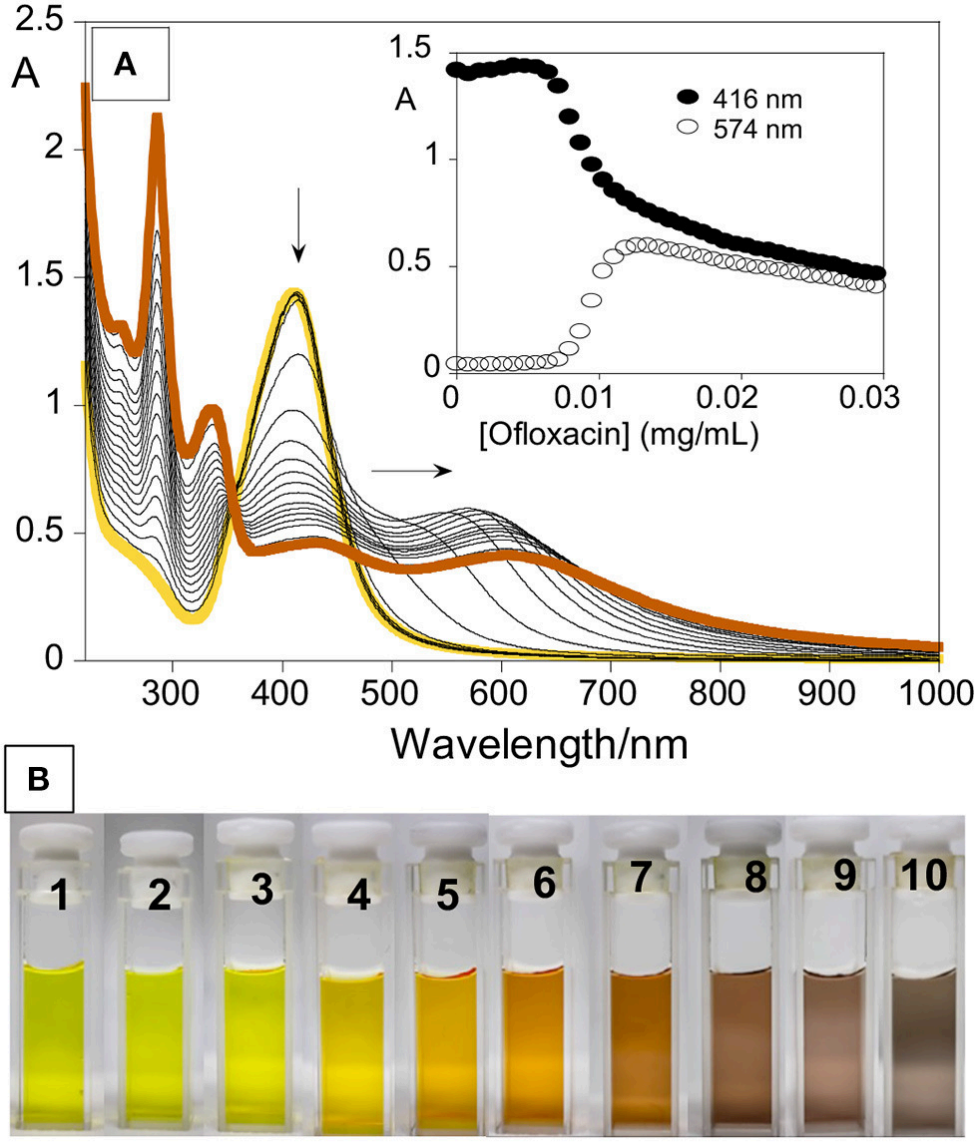
**(A)** Spectrophotometric titration of AgNPs with increasing amounts of ofloxacin in water. Inset represents the absorption as a function of ofloxacin concentration (mg/mL) at 416 nm and 574 nm. **(B)** Naked-eye colorimetric response of AgNPs with increasing amount of ofloxacin; 1–0, 2–2, 3–14, 4–22, 5–26, 6–30, 7–34, 8–38, 9–42, 10–46 μL from a stock solution of ofloxacin (1 mg/mL).

During the titration, a redshift of the resonance plasmon band from 400 to 574 nm, was observed (Figure 2A), but the nanoparticles did not stabilize and precipitate after 2 h. The broadening and shift toward lower energies of the absorption band are very reasonable due to the nanoparticle surface exchange of the labile citrate molecules with ofloxacin, via its carboxylic group. Ofloxacin is a large organic molecule that is not able to efficiently protect the metal surface of the NP and, as well-known and documented when the surface coverage is inefficient metal particles aggregate and precipitate. The evolution of the AgNPs@ofloxacin interaction was denoted by a clearly visible change in the color of the solution (Figure 2B): from the characteristic yellow color of a spherical AgNPs solution (Figure 2B; 1-3) to orange (Figure 2B; 4-6), brown (Figure 2B;7-9), and finally gray (Figure 2B; 10).

**Figure 3:**
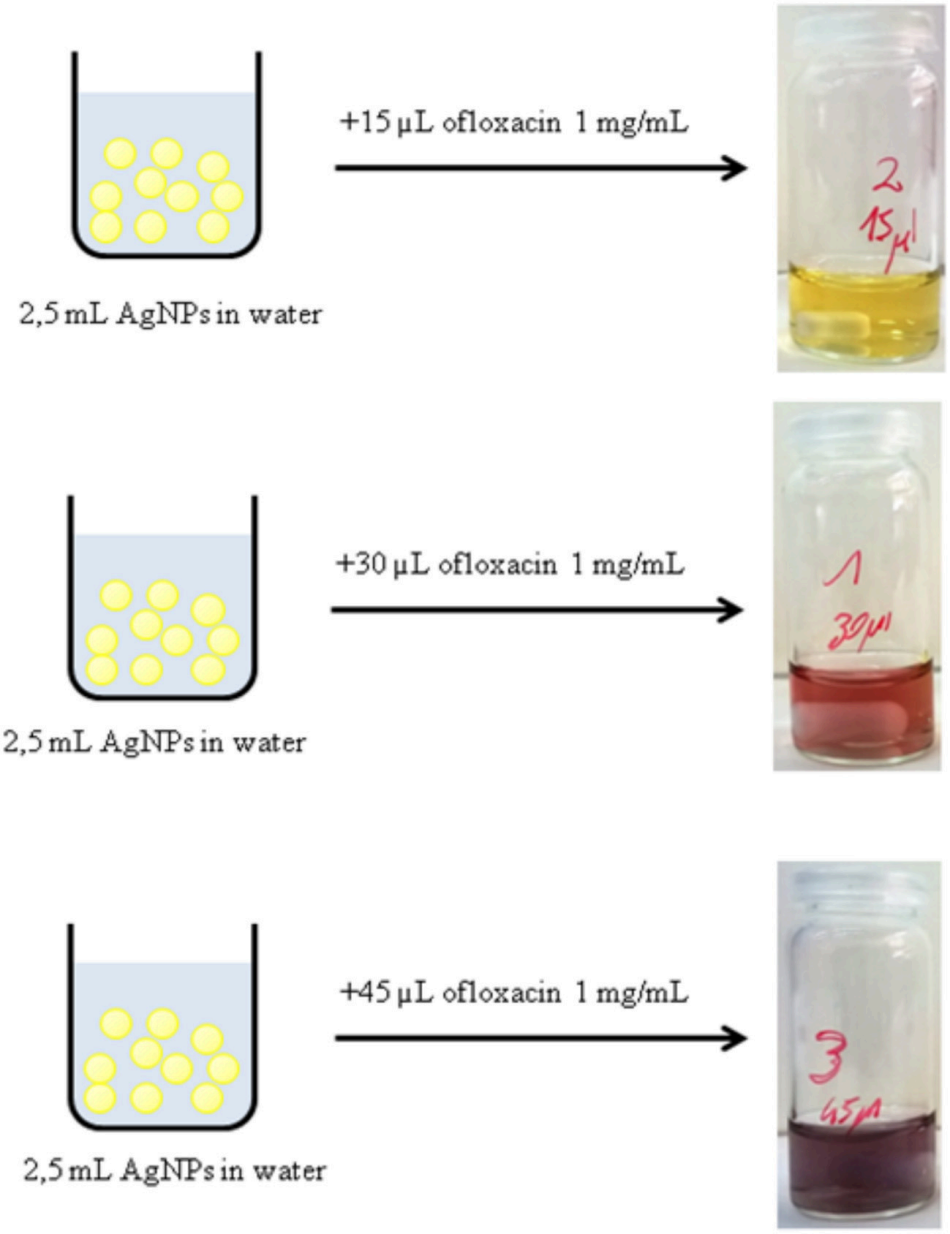
Immediate color change of a solution of AgNPs after the addition of different amounts of a solution 1 mg/mL of ofloxacin.

Following abovementioned results, the AgNPs were incubated for 24 h with different amounts of 1 mg/mL ofloxacin solution (30, 15, 45, 78 μL). Immediately after the addition of the antibiotic solution a change in the color was observed (Figure 3). After 24 h stirring at r.t. and in dark environment conditions all solutions were colorless and transparent and a dark precipitate was formed, the nanoparticles aggregated and precipitated.

### Synthesis and characterization of MSNPs and AgMSNPs

**Figure 4:**
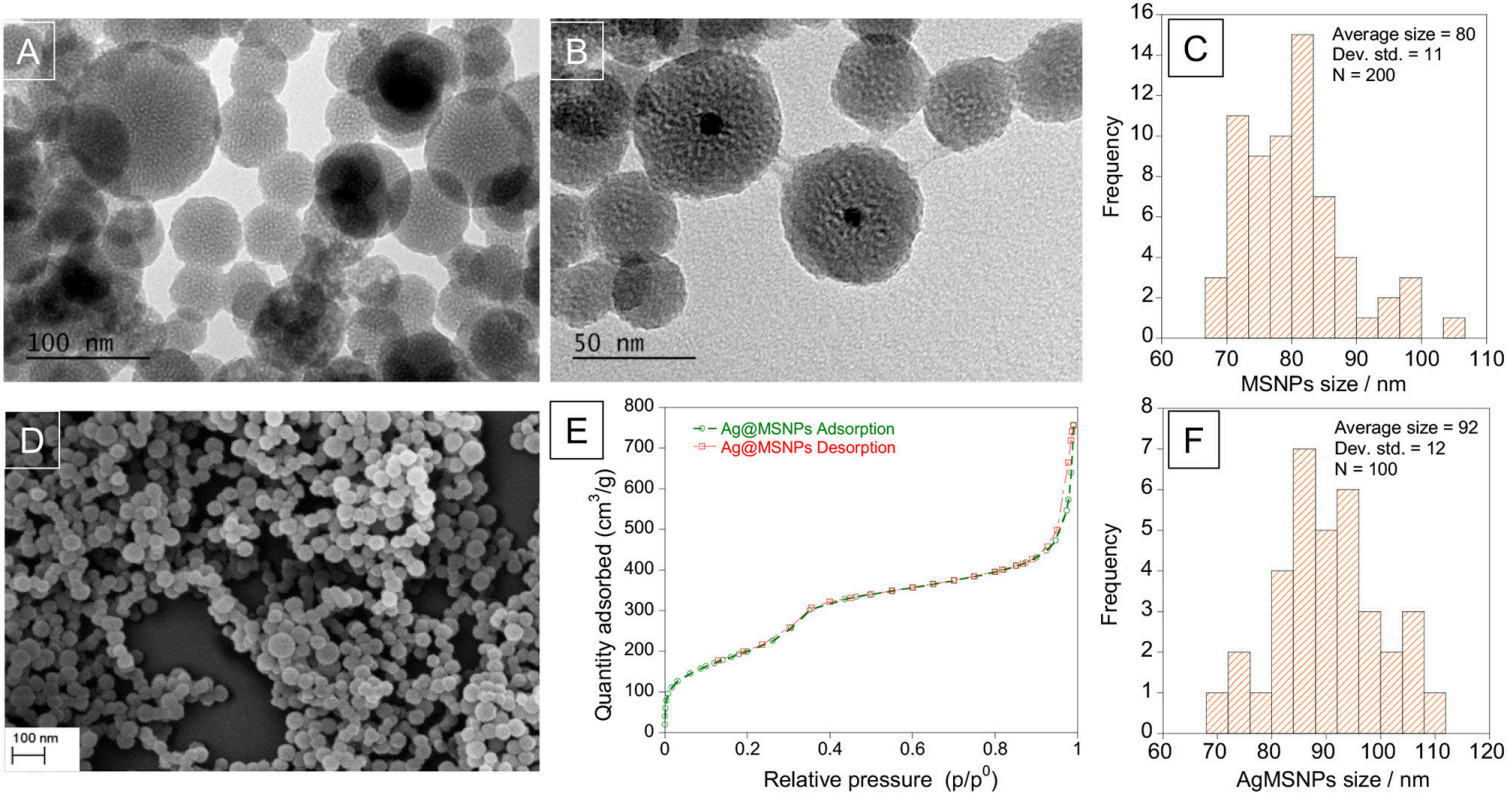
TEM images of mesoporous silica nanoparticles, MSNPs **(A)** and silver doped mesoporous silica nanoparticles, AgMSNPs **(B)**, SEM image **(D)**, and Nitrogen isotherms **(E)** of AgMSNPs. Size distribution histograms of MSNPs **(C)** and AgMSNPs **(F)**.

Silver mesoporous nanoparticles were obtained by encapsulation of AgNPs inside a silica matrix to generate mesoporous nanoparticles. In this synthesis, TEOS was used as a silica source, CTAB as template and cationic surfactant, ethylene glycol as stabilizer and NaOH as a morphological agent. This approach was based on MCM-41 synthesis (Kresge et al., 1992; Oliveira et al., 2018), and different amounts of TEOS (250 μL−1mL) were tested in order to find the most stable system. As a control, mesoporous silica nanoparticles (MSNPs) were also obtained but without the silver nanoparticle core. As expected both systems were of spherical shape: MSNPs and AgMSNPs were obtained as a powder, and were characterized by dynamic light scattering (size and zeta potential), TEM, SEM, ICP, and N_2_ isotherms (Figure 4).

Concerning the MSNPs, they were characterized from a zeta potential point of view to choose the most stable batch to proceed with encapsulation of ofloxacin. The measure was conducted solubilizing 1 mg of nanoparticles in 1.5 mL of DMSO and using a dip cell for the measurement of the zeta potential. The average value is −30.8 ± 0.3 mV. TEM images reveal high porosity of the MSNPs, as can be seen by the white dots, showing an average diameter of 80 ± 11 nm (Figure 4C).

In the synthesis of AgMSNPs different quantities of TEOS were used to find the amount that leads to the most stable particles, for subsequent ofloxacin encapsulation. The AgMSNPs obtained were characterized by DLS measurements for size and zeta potential (see Table 2).

**Table 2:** Results for the size and ζ potential characterization of AgMSNPs.

**TEOS amount**	**Hydrodynamic diameter (nm)**	**PDI**	**Zeta potential (mV)**
250 μL	267 ± 47	0.89 ± 0.18	−36.67 ± 1.02
500 μL	433 ± 181	0.9 ± 0.1	−44.12 ± 1.64
750 μL	416 ± 251	1	−41.7 ± 2.08
1 mL	633 ± 265	0.83 ± 0.16	−40.4 ± 1.99

The AgMSNPs synthesized with 500 μL of TEOS turned out to be the most stable, presenting the most negative zeta potential (−44 mV). AgMSNPs (500 μL TEOS) were also covered with PDMAC, a polyelectrolyte, to improve their solubility and reduce their aggregation. Size and zeta potential measurements were conducted; the results revealed a decrease in size (from 430 ± 180 nm to 270 ± 40 nm) and PDI (from 0.9 ± 0.1 to 0.24 ± 0.01), and as expected a total inversion of the zeta potential (from −44.1 ± 1.6 to +47.7 ± 0.9 mV), confirming the total surface coverage by the polyelectrolyte and the higher stability in water. As can be seen in TEM images AgMSNPs have a silver core with a silica shell, having a total average radius of 92 ± 12 nm (Figure 4F). ICP confirmed the silver core nature and the amount of 2,879 ppm of silver was determined, with a ratio of 2.8 mg of Ag per gram of nanoparticles. The size and the spherical shape were also confirmed by SEM studies (Figure 4D).

Moreover, AgMSNPs showed nitrogen adsorption-desorption type IV isotherms of ordered mesoporous with an adsorption step behavior at p/p^0^ around 0.30–0.35. Surface area, pore size and pore volume were assessed using Brunauer-Emmett-Teller (BET) and Barrett-Joyner-Halenda (BJH) theories (data in Table 3). From these curves pore volumes of 1.2 cm^3^/g were calculated by the BJH model for AgMSNPs, on the adsorption branch of the isotherm. Through the application of the BET model, the average pore width and surface area were also estimated and resulted in 52 Å and 759 m^2^/g, respectively. Besides, the presence of mesoporosity in the synthesized systems, the BET surface areas are slightly lower than the traditional MCM-41 mesoporous nanoparticles [1110 ± 2 m^2^/g; (Huang et al., 2014)], which is compatible with the presence of the silver nanoparticles forming the core of these nanosystems.

**Table 3:** BET and BJH porosimetry measurements for Ag@MSNPs (500 μL TEOS) AgMSNPs.

**SURFACE AREA**	
BET Surface Area	759.4336 m^2^/g
BJH Adsorption cumulative surface area of pores between 17,548 Å and 1 585,580 Å diameter	878.015 m^2^/g
BJH Desorption cumulative surface area of pores between 17,000 Å and 3 000,000 Å diameter	882.0866 m^2^/g
**PORE VOLUME**	
BJH Adsorption cumulative volume of pores between 17,548 Å and 1 585,580 Å diameter	1.226786 cm^3^/g
BJH Desorption cumulative volume of pores between 17,000 Å and 3 000,000 Å diameter	1.231038 cm^3^/g
**PORE SIZE**	
Adsorption average pore diameter (BET)	52.1688 Å
BJH Adsorption average pore diameter	55.889 Å
BJH Desorption average pore diameter	55.824 Å

### Encapsulation of ofloxacin into MSNPs and AgMSNPs

Ofloxacin loading was evaluated after 24 h incubations of 1 mL of a solution of ofloxacin in DMSO and PBS pH 7.4 at various concentrations with approximately 4.5–5 mg of MSNPs or AgMSNPs. After incubation, the nanoparticles were centrifuged, and the supernatant was collected to perform absorption measurements and calculate the encapsulation percentages accordingly with equation (2) (section Encapsulation and functionalization of ofloxacin into nanoparticles; Table 4).

**Table 4:** Encapsulation % of ofloxacin in MSNPs and AgMSNPs in DMSO (1), (2), and PBS pH 7.4 (3).

**[Ofloxacin](M)**	**Encapsulation % in MSNPs (1)**	**Encapsulation % in AgMSNPs (2)**	**Encapsulation % in AgMSNPs (3)**
1 × 10^−5^	~10	~35	~84
1 × 10^−4^	~23	~29	~50
5 × 10^−4^	~34	–	–
7 × 10^−4^	~30	–	–
1 × 10^−3^	~30–89	~29	38
1 × 10^−2^	–	~35	~9

**Figure 5:**
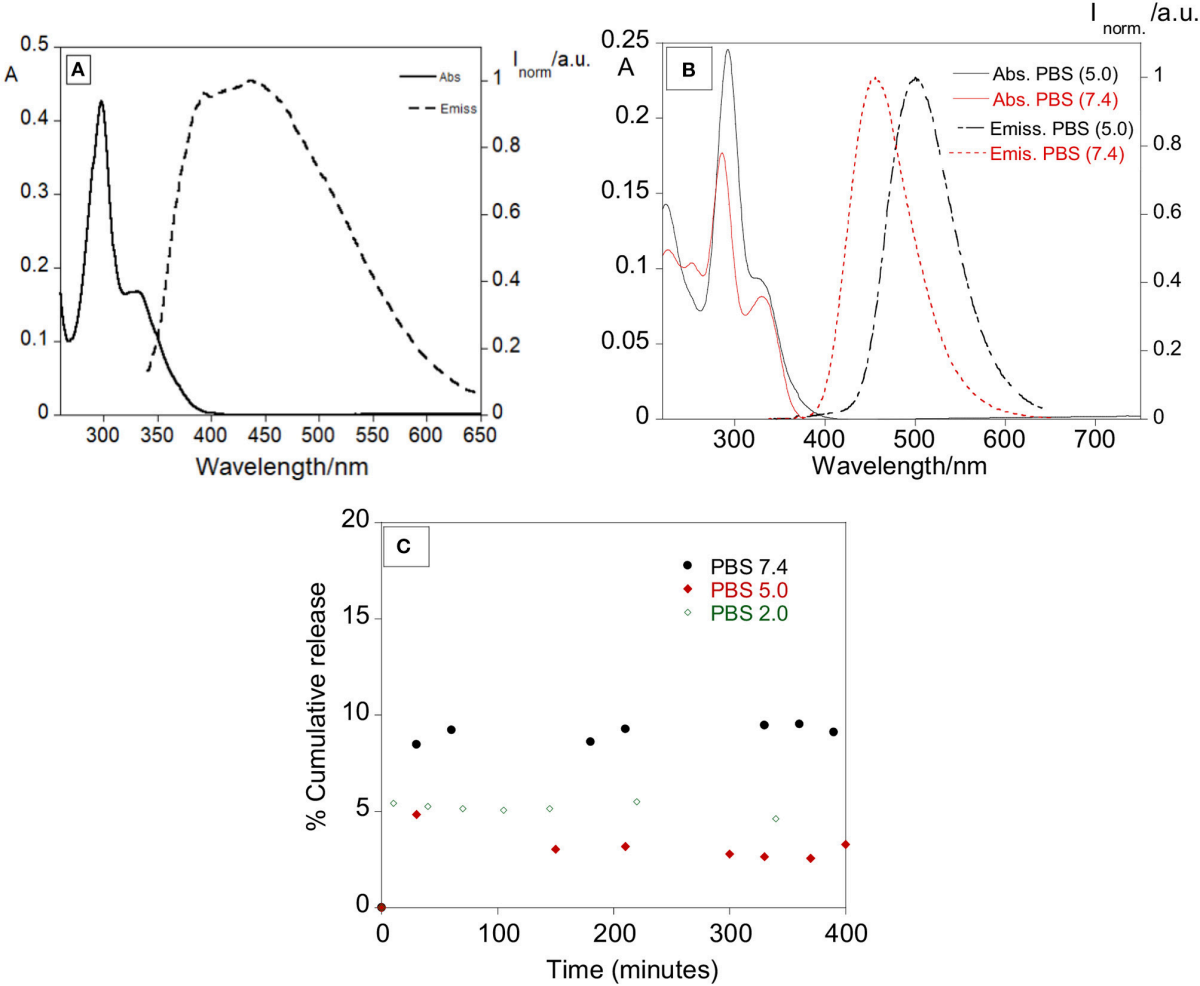
**(A)** Absorption and emission spectra of ofloxacin in DMSO (λexc = 330 nm). **(B)** Absorption and emission spectra of ofloxacin in PBS at pH 5.0 and pH 7.4. (λexc = 330 nm). [Ofloxacin] = 1 × 10^−5^ M, r.t. **(C)** Percentage of cumulative release with time of ofloxacin in PBS pH 7.4, PBS pH 5.0, and PBS pH 2.0.

As a control, a solution of free ofloxacin 1 × 10^−5^ M was characterized in DMSO, PBS pH 7.4 and PBS pH 5.0 (Figure 5). Absorption, emission and excitation spectra of ofloxacin were collected at r.t and in DMSO it presents an absorption maximum at 300 nm with a shoulder at ca. 350 nm and an emission maximum at 450 nm (Figure 5A).

Concerning further applications in biological media, the ofloxacin was characterized by absorption and emission in PBS pH 7.4 and PBS pH 5.0. In PBS pH 5.0 (Figure 5B) ofloxacin exhibits an absorption band with the maximum centered at 293 nm and a shoulder at 330 nm, in the emission spectra the maximum is at 500 nm.

In PBS pH 7.4 (Figure 5B) ofloxacin exhibits an absorption band slightly different from the one in PBS pH 5.0, with the maximum centered at 286 nm and a shoulder at 300 nm, in the emission spectra the maximum is also shifted to 456 nm.

It is possible to notice a variation both in the absorption and emission bands, suggesting an influence of the solvent and of the pH on the photochemical behavior of ofloxacin.

Concerning the MSNPs, the encapsulation values are quite uniform, in the range of 10^−4^ M, with % E values around 30%. Regarding the AgMSNPs in a general way, the trend for the encapsulation performed in DMSO is not regular, conversely, when the encapsulations were performed in PBS the trend shows a regular progress with a bigger amount of ofloxacin encapsulated for lower initial concentrations. This can be attributed to the fact that the drug is poorly soluble in aqueous environment, showing then the tendency to migrate to the pores of the silica mesoporous nanoparticles.

MSNPs with ofloxacin were also characterized with DLS measurement for the size (hydrodynamic diameter: 243 ± 208 nm, PDI = 1) and zeta potential (−27.7 ± 1.6 mV). These results indicate that with the addition of ofloxacin, the nanoparticles were slightly destabilized, if compared to the crude MSNPs.

Some preliminary tests for the release of ofloxacin were performed at 37°C in PBS at various pH to simulate a physiological media. The release was followed mainly by absorption measurements, recording the absorption of the supernatant over approximately 20 hours. The total observed release % was of 9.5, 4.9, and 5.4% for pH 7.4, 5.0, and 2.0, respectively. Despite the lower release percentage observed in PBS (*in vitro* simulation), it was clear the antibacterial effect of our nanosystem in the strains used, showing an effective synergetic effect between the silver core and the antibiotic.

### Synthesis of dye-doped SNPs and ofloxacin functionalization

The synthesis of the dye-doped SNPs, as well as, the covalent ofloxacin functionalization was performed according to the protocol described in the experimental section.

#### Photophysical characterization

**Figure 6:**
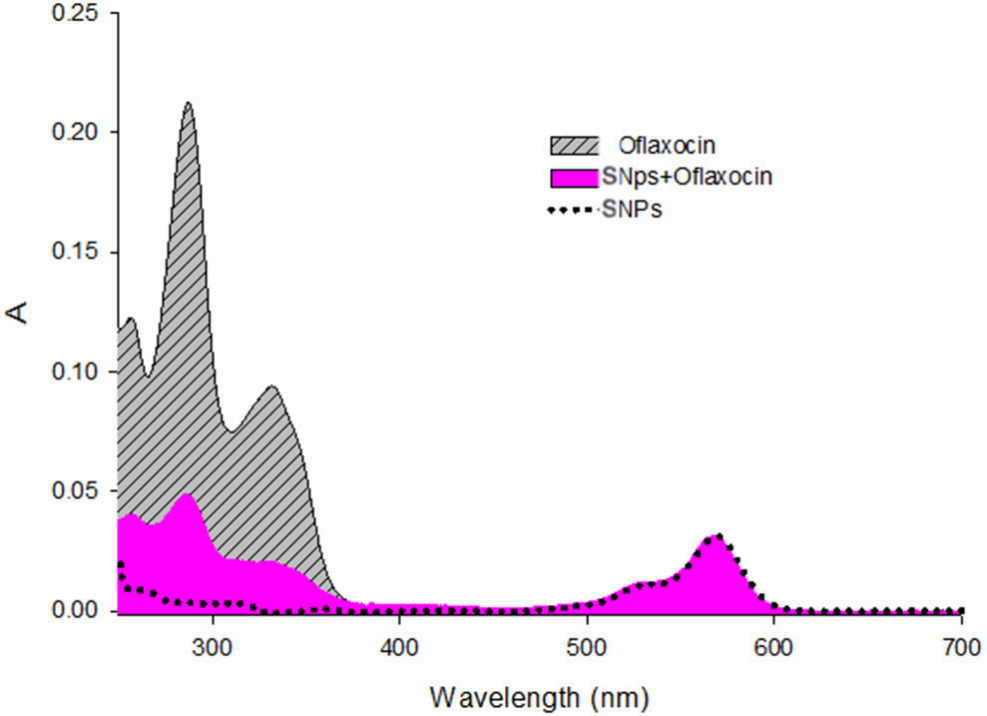
Absorption spectra of Ofloxacin [9.3μM], SNPs (nanoparticles) [0.18μM], and SNPs+Ofloxacin [0.18 μM] in PBS pH 7.4.

To quantitatively assess the functionalization of the nanoparticle surface with ofloxacin, absorption spectra were performed for both ofloxacin and functionalized SNPs in PBS 0.01 M pH 7.4 (Figure 6). Assuming that ofloxacin, when conjugated to the nanoparticles presents the same absorption than the free drug, we could estimate that the average number of ofloxacin molecules per NP is 13.

DLS measurements confirm the narrow distribution of the dispersion of the NPs with a hydrodynamic diameter around 25 nm.

**Figure 7:**
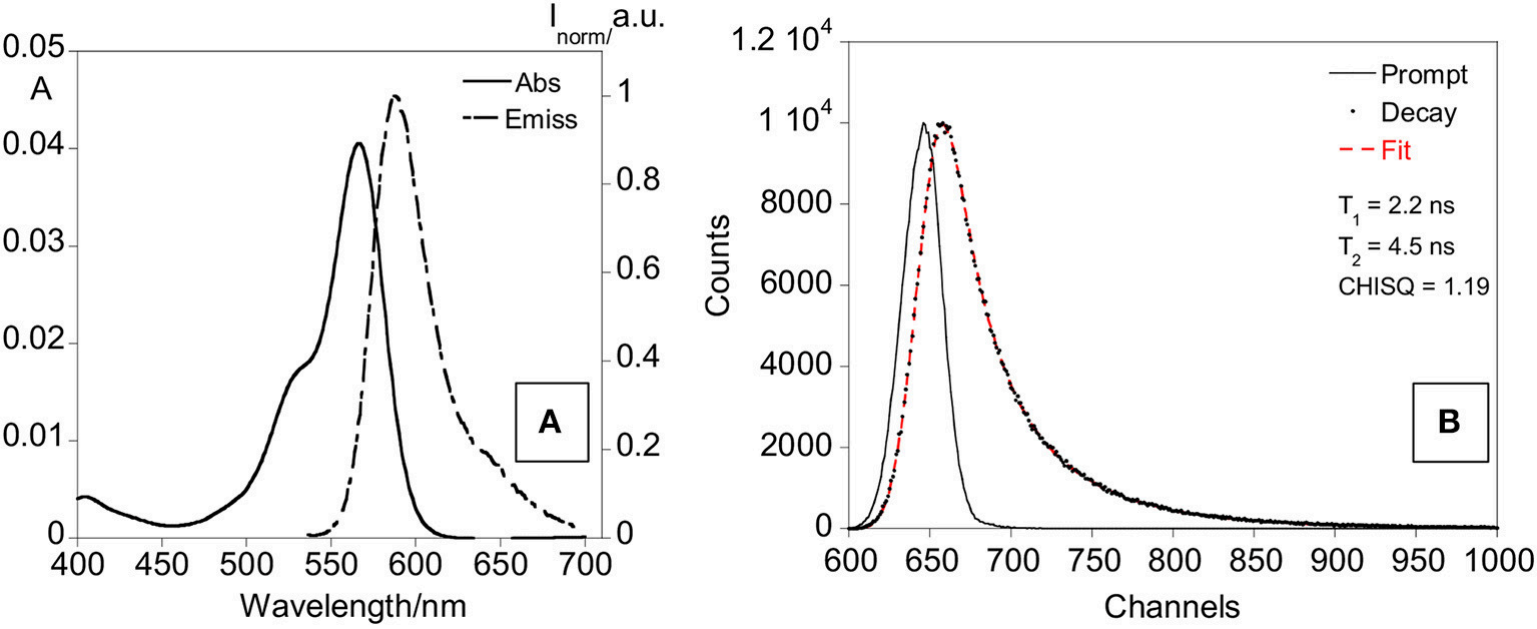
Absorption and emission spectra **(A)** and lifetime decay **(B)** of rhodamine-doped SNPs in water.

The final nanoparticles (dye (rhodamine)-doped SNPs@ofloxacin) were characterized by recording absorption and emission spectra, lifetimes, DLS and Z-potential, also the fluorescence quantum yield was calculated. The absorption and emission spectra show bands centered at 569 and 595 nm, respectively, characteristic of the rhodamine dye (Figure 7). A fluorescence quantum yield of 0.21 was determined for the rhodamine-doped silica nanoparticles, using as standard the rhodamine. Concerning the lifetime measurements, the exponential decay was fitted for two species with values of 2.2 ns, 4.5 ns, and a chi sq of 1.19. The standard rhodamine compound presents a lifetime of 2.8 ns.

**Figure 8:**
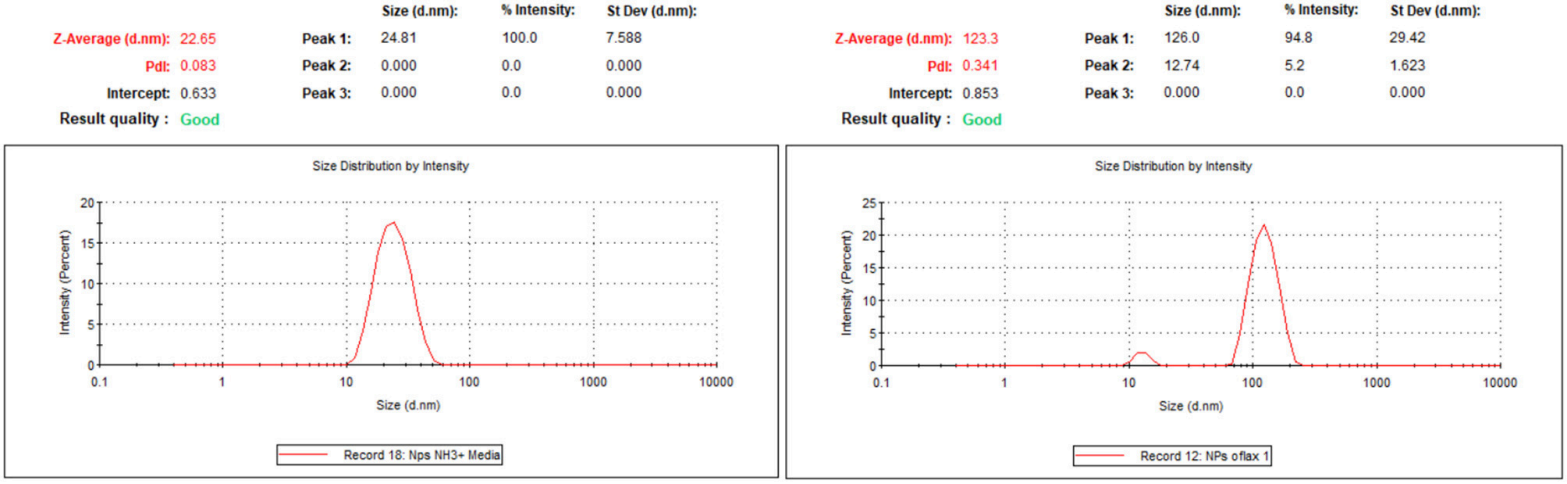
DLS measurements for SNPs-NH_2_ and SNPs-Ofloxacin on the right both in PBS at pH 7.4.

Z-potential values are −4.6 mV for SNPs-NH_2_ and −10.6 mV for SNPs-Ofloxacin; DLS measurements show once again the functionalization of the SNPs surface: the hydrodynamic radius for SNPs is around 20 nm while for the functionalized SNPs is around 100 nm (Figure 8).

### Bactericidal activity assays

Samples of MSNPs and AgMSNPs with different concentrations of ofloxacin (Oflx) and dye-doped SNPs decorated with ofloxacin were all tested to assess their bactericidal activity.

Minimum inhibitory concentration (MIC) and minimum bactericidal concentration (MBC), against both susceptible and resistant *E. coli* and *S. aureus* strains, were evaluated according to the procedures described in the experimental section (section Antibacterial Susceptibility Test). The results for all the nanoparticle samples that represent new formulations are listed in Table 5.

**Table 5:** Minimum inhibitory concentration and minimal bactericidal concentration under nanoparticle plus antibiotic system.

**Nanoparticle description**	**Strain**	**MIC (μg/mL)**	**MBC (μg/mL)**
AgMSNPs ofloxacin ~ 50 μM	*E. coli* K12 ATCC 29425	10	25
	*E. coli* C999 (CTX-M-15)	50	75
	*S. aureus* ATCC 25923	5	25
	*S. aureus* C5932 (MRSA CC398)	100	200
AgMSNPs ofloxacin ~ 8 μM	*E. coli* K12 ATCC 29425	25	25
	*E. coli* C999 (CTX-M-15)	50	75
	*S. aureus* ATCC 25923	10	25
	*S. aureus* C5932 (MRSA CC398)	100	200
MSNPs ofloxacin ~ 750 μM	*E. coli* K12 ATCC 29425	10	25
	*E. coli* C999 (CTX-M-15)	100	300
	*S. aureus* ATCC 25923	10	25
	*S. aureus* C5932 (MRSA CC398)	200	ND
MSNPs ofloxacin ~ 650 μM	*E. coli* K12 ATCC 29425	10	25
	*E. coli* C999 (CTX-M-15)	200	500
	*S. aureus* ATCC 25923	50	50
	*S. aureus* C5932 (MRSA CC398)	300	ND
MSNPs ofloxacin ~ 270 μM	*E. coli* K12 ATCC 29425	25	50
	*E. coli* C999 (CTX-M-15)	200	500
	*S. aureus* ATCC 25923	50	75
	*S. aureus* C5932 (MRSA CC398)	500	ND
Dye-doped SNPs Ofloxacin ~ 20 μM	*E. coli* K12 ATCC 29425	100	ND
	*E. coli* C999 (CTX-M-15)	ND	ND
	*S. aureus* ATCC 25923	ND	ND
	*S. aureus* C5932 (MRSA CC398)	ND	ND
AgMSNPs	*E. coli* K12 ATCC 29425	25	50
	•*E. coli* C999 (CTX-M-15)	50	100
	*S. aureus* ATCC 25923	10	50
	*S. aureus* C5932 (MRSA CC398)	100	300
	*E. coli* K12 ATCC 29425	500	ND
MSNPs	•*E. coli* C999 (CTX-M-15)	ND	ND
	*S. aureus* ATCC 25923	500	ND
	*S. aureus* C5932 (MRSA CC398)	ND	ND
DMSO	*E. coli* K12 ATCC 29425	ND	ND
	•*E. coli* C999 (CTX-M-15)	ND	ND
	*S. aureus* ATCC 25923	ND	ND
	*S. aureus* C5932 (MRSA CC398)	ND	ND
Free ofloxacin	*E. coli* K12 ATCC 29425	10	10
	*E. coli* C999 (CTX-M-15)	ND	ND
	*S. aureus* ATCC 25923	5	5
	*S. aureus* C5932 (MRSA CC398)	ND	ND

*MIC, minimum inhibitory concentration; MBC, minimal bactericidal concentration*.

The tests show that the best performance is obtained with the AgMSNPs@Oflx samples. All the other systems are not effective against the *S. aureus* C5932, the MSNPs@Oflx systems are only able to inhibit their growth but not to kill the bacteria. The AgMSNPs@Oflx samples can inhibit the growth and kill all of the bacterial strains. This interesting result can be explained hypothesizing a synergic effect of the antibiotic and the Ag core of the nanoparticles, as silver has antibacterial properties itself. The strain that was more susceptible was the *S. aureus* ATCC 25923, and the lowest values for MIC and MBC among all the samples tested were again presented by the AgMSNPs@Oflx sample (MIC = 5 and 10 and MBC = 25 μg/mL for [Oflx] = 50 and 8 μM). The results also showed that the inhibitory effects of ofloxacin are highly dependent on its concentration as already mentioned and as attended.

As a control, non-loaded AgMSNPs and MSNPs, DMSO and free ofloxacin were tested in the presence of the same bacterial strains (Table 1). As expected, free AgMSNPs show a certain rate of bactericidal activity proving the effectiveness of the silver cores, while MSNPs and DMSO exhibit no antibacterial activity (Liong et al., 2009; Xu et al., 2009; Xiu et al., 2012; Yu et al., 2015; D'Agostino et al., 2017). Moreover, it is interesting to notice the difference in values of MBC between free AgMSNPs (MBC = 50 μg/mL) and AgMSNPs@Oflx (MBC = 25 μg/mL) loaded with the lowest ofloxacin concentration, in the presence of strain *S. aureus* ATCC 25923. These results prove the synergistic antibacterial effect of the silver nanoparticles combined with ofloxacin. On the other hand, this could be also considered together with the high surface/mass ratio typically present in nanomaterials: the smaller the particles, the higher the metallic surface exposed, and subsequently higher microbicidal effect can be expected (El Badawy et al., 2011).

Ofloxacin was also tested alone, in the same concentrations it has in the samples when loaded in the nanoparticles (Table 5). As a result, in the case of *E. coli* K12 ATCC 29425 and *S. aureus* ATCC 25923 only their growth was inhibited and no bacteria were killed, again proving the importance of the association between antibiotics and silver nanoparticles.

The dye-doped SNPs functionalized with Ofloxacin did not show a significant antibacterial activity against the tested bacterial strains, suggesting again the crucial role of silver in increasing the therapeutic efficacy and maybe a role of the silica shell in the bacterial uptake of the nanoparticles. In Gram^−^negative bacteria AgNPs toxicity may arise directly from physical processes caused by nano-objects, like disruption of cell membrane and penetration of NPs into the cytoplasm with subsequent Ag^+^-DNA binding or interaction with bacterial ribosome (Xu et al., 2004; Yamanaka et al., 2005).

## Conclusions

Mesoporous based nanocarriers with and without a silver core, MSNPs and, AgMSNPs, as well as, dye (rhodamine)-doped silica nanocarriers were successfully obtained and characterized. The MSNPs and AgMSNPs showed a negative zeta potential of −30.8 ± 0.3 mV and −44.1 ± 1.6 mV, as well as, diameter sizes of 80 ± 11 and 92 ± 12 nm, respectively. Regarding the rhodamine-doped silica nanoparticles a hydrodynamic ratio of 25 nm was obtained and a zeta potential of −4.6 mV.

The antibiotic ofloxacin was encapsulated and functionalized in all synthesized nanocarriers and the study of their antibacterial properties performed against *S. aureus* and *E. coli*.

The best performance was obtained with the AgMSNPs@ofloxacin samples, being able to inhibit the growth and kill all bacterial strains. The strain *S. aureus* ATCC 25923 was the most susceptible to the AgMSNPs@ofloxacin nanocarrier, presenting the lowest values for MIC (5 μg/mL), and MBC (25 μg/mL) for an ofloxacin concentration of 50 μM. The results also prove a synergistic antibacterial effect of the silver nanoparticles combined with ofloxacin.

## Author contributions

LP, CL, and EO design and supervised the project. SN, JF-L, VS, EO, ER, and BR-G performed the experiments. EO, SN, LP, NZ, JF-L, CL, and GI analyzed the results and wrote the paper. EO, LP, CL, JC, GI, PP, NZ, and CT provide the resources related to the project. EO, LP, NS, CL, and JC financed the project. All authors reviewed the manuscript and approved the final version.

### Conflict of interest statement

The authors declare that the research was conducted in the absence of any commercial or financial relationships that could be construed as a potential conflict of interest.
